# The Convergence of Fibrosis and Granulomatous Inflammation: A Case Series on the Diagnostic and Therapeutic Challenges of Systemic Sclerosis and Sarcoidosis Overlap Syndrome

**DOI:** 10.3390/diagnostics16121871

**Published:** 2026-06-16

**Authors:** Maria Laura Groșeanu, Ionela Belaconi, Ionela Mihaela Erhan, Daniela Anghel

**Affiliations:** 1 Faculty of General Medicine, Carol Davila University of Medicine and Pharmacy, Bld Eroii Sanitari nr 8, 050474 Bucharest, Romania; ionela.belaconi@yahoo.com; 2 Sfanta Maria Clinical Hospital, Bld Ion Mihalache 37-39, 0111172 Bucharest, Romania; 3 Marius Nasta National Pneumology Institute, Sos Viilor 90, 050159 Bucharest, Romania; 4 Carol Davila Military Central Hospital, 050474 Bucharest, Romania; nely_erhan@yahoo.com (I.M.E.); daniela.anghel@prof.utm.ro (D.A.); 5 Department of Medico Surgical and Prophylactic Disciplines, Titu Maiorescu University, Gheorghe Pătrașcu Street 67A, 031593 Bucharest, Romania

**Keywords:** sarcoidosis, systemic sclerosis, autoimmune conditions, interstitial lung disease, treatment challenges

## Abstract

The coexistence of systemic sclerosis and sarcoidosis is very rare. Both are systemic autoimmune diseases with lung involvement but different pathogenesis. In contrast to findings of mid- to upper-lobe interstitial lung disease (ILD) that may occur with hilar lymphadenopathy in sarcoidosis, the most common lung manifestation of systemic sclerosis is lower-lobe ILD, which is typically characterized by a non-specific interstitial pneumonia pattern. This distinction in lung involvement between the two diseases is crucial due to the need for different therapeutic approaches. We present a series of three overlap cases: two with sarcoidosis onset before the diagnosis of systemic sclerosis and another with systemic sclerosis onset before the diagnosis of sarcoidosis. Three cases of concomitant sarcoidosis and systemic sclerosis are reviewed, including the pathophysiology of each disease and the shared pathways leading to the development of both conditions. Systemic sclerosis–sarcoidosis overlap is a high-risk phenotype. Early recognition and a personalized, aggressive therapeutic approach are essential to alter the natural course of these two converging fibrotic and granulomatous processes.

## 1. Introduction

Sarcoidosis is a multisystem disease of unknown etiology that is defined by multiple sterile noncaseating granulomas [1]. Almost any organ can be affected, but the lungs and mediastinal lymph nodes are by far the most affected (>90% of patients), manifesting as bilateral hilar lymphadenopathy and/or interstitial lung disease (ILD) that is visible on lung imaging [2]. Sarcoidosis may be associated with other autoimmune diseases such as Hashimoto’s thyroiditis, systemic lupus erythematosus, and rheumatoid arthritis, but its association with systemic sclerosis (SSc) is rare [3,4].

ILD is a common feature in both diseases, and clinical, radiological, laboratory, and bronchoscopic differentiation may be difficult. Nevertheless, thorax computed tomography (CT) can reveal distinct features. SSc-ILD typically shows a non-specific interstitial pneumonia (NSIP) pattern with ground-glass opacities, reticulation opacities, traction bronchiectasis, and honeycomb cysts mainly in the lower lung zones [5]. Other, less common HRCT patterns in SSc-ILD are: usual interstitial pneumonia (UIP) pattern characterized by reticular abnormalities, honeycombing, traction bronchiectasis, with basal and peripheral predominance and organizing pneumonia (OP) pattern characterized by patchy consolidations, often peripheral or peribronchial [5]. In contrast, the lung manifestations of sarcoidosis include bilateral hilar and mediastinal lymphadenopathy but may also involve beading or irregular thickening of the bronchovascular bundles, bronchial wall thickening, nodules along the bronchi and blood vessels, subpleural lesions, and mid- to upper-lobe-predominant ground-glass opacities [6,7].

The granulomas found in sarcoidosis have a typical histological appearance. In contrast, a sarcoid-like reaction refers to a granulomatous response that resembles sarcoidosis but occurs as a secondary reaction to another underlying cause, such as infections, malignancies, drugs, or other inflammatory conditions. These reactions tend to be localized near the primary disease site and are often discovered incidentally during the evaluation of other diseases. The granulomas look similar to those in sarcoidosis but arise as part of a secondary immune response. Management focuses on treating the underlying cause, and sarcoid-like reactions usually improve once the triggering factor is controlled [8].

The differentiation of ILD related to each disease is important for prognosis and therapy. Whereas SSc-ILD may be progressive and fatal, lung involvement associated with sarcoidosis is more benign [9] and is treated based on the severity of symptoms. For asymptomatic patients with stage 0 or I on chest x-ray, therapy is not likely to offer benefits. Corticosteroids (GCs) remain the initial drug of choice for treatment of parenchymal lung diseases. The starting dosage is 20–40 mg prednisone or its equivalent, which is tapered at 3–6 months to 10 mg of prednisone per day or less. The addition of a steroid-sparing agent, such as methotrexate, azathioprine, or even an anti-TNF, can be individually considered based on the evolution of the disease. In more advanced lung involvements, corticosteroids (CSs) and immunosuppressive (IS) drugs are used, while antifibrotics are considered in cases of a progressive phenotype [9]. However, for SSc-ILD, the treatment decision is influenced by baseline and clinically meaningful changes in PFT values, the extent of ILD or fibrosis on HRCT, the presence of respiratory symptoms, and potential risk factors for progression [10]. Therapeutic options include IS drugs such as mycophenolate and cyclophosphamide, and antifibrotic therapies such as nintedanib can be used alone or in combination with mycophenolate [11]. Several open-label studies and a meta-analysis involving 20 studies support the beneficial effects of off-label rituximab. The FDA has also approved the use of tocilizumab for SSc-ILD [12]. Finally, there is a strong recommendation against the use of GCs for SSc-ILD treatment [11,12].

Although the pathogenesis of sarcoidosis and SSc is complex, some differences in immune system activation can be underlined. Sarcoidosis is a T-helper 1 (Th1)-mediated disease that results in noncaseating epithelioid cellular granuloma formation [13]. CD4+ T-lymphocytes have been identified in the tissues of patients with sarcoidosis and appear to be primarily concentrated within the granulomatous lesion [14]. The levels of the Th1 cytokines interferon (IFN)-λ and interleukin (IL)-2 are elevated in sarcoidosis, while the levels of the T-helper 2 (Th2) cytokines IL-4 and IL-5 decrease [15]. The complex pathophysiology of SSc varies depending on the stage of the disease, with endothelial cell dysfunction being the initial event, followed by immune system activation [16]. The early and active stages of SSc involve the Th2 pathway, with disease progression believed to have been caused by damage to the endothelial lining in small blood vessel walls, leading to impaired circulation and subsequent tissue hypoxia and collagen deposition from activated resident fibroblasts [17]. While the pathophysiological foundation of SSc and sarcoidosis is significantly different, commonalities with regard to cytokines have been demonstrated; for example, IL-6 was found to be elevated in the bronchoalveolar lavage fluid of patients with active sarcoidosis [17], as well as those with SSc [18,19]. Fibrosis is also a pathologic attribute shared by both SSc and sarcoidosis, with multiorgan involvement in SSc and a more localized lung fibrosis in patients with sarcoidosis [19,20]. In sarcoidosis, a promoter polymorphism, −765 G > C, has been demonstrated to be associated with lung fibrosis onset [21]. Both conditions share high TGF-β1 expression. In patients with sarcoidosis, the levels of TGF-β1 in peripheral blood lymphocytes and bronchoalveolar cells are increased [22]. The cytokine TGF-β1 induces COX-2 expression through the Smad signaling pathways and is known to drive fibrosis in systemic sclerosis [23]. TGF-β2 and TGF-β3 gene polymorphisms have been implicated in cutaneous fibrosis associated with SSc [24], and similar findings have been reported by Kruit and colleagues in a four-year follow-up study of sarcoidosis patients who developed pulmonary fibrosis when bearing a genetic variation in TGF-β3 [25].

## 2. Clinical Significance

The purpose of our work is to explore and analyze the complex interplay between fibrosis and granulomatous inflammation in patients exhibiting features of both SSc and sarcoidosis. Through a detailed case series, the article aims to highlight the diagnostic difficulties and therapeutic challenges encountered in recognizing and managing this rare overlap syndrome, ultimately providing insights to improve clinical outcomes and guide future research.

## 3. Case Presentation

### 3.1. Case 1

In 2014, a 31-year-old Caucasian female presented with a three-year history of Raynaud’s phenomenon, arthralgias of the small joints of the hands, and progressive skin thickening involving the fingers, dorsal hands, and face. Immunological screening revealed high titers of anti-Scl70 antibodies (>200 UI/mL) and no other specific autoantibodies. Nailfold capillaroscopy demonstrated an “early” scleroderma pattern. Since initial lung function tests (PFTs) were within the normal limits, a baseline chest CT was not performed. Based on ACR/EULAR 2013 classification criteria for SSc [26] the patient was diagnosed with systemic sclerosis with limited cutaneous involvement, and treatment with calcium channel blockers (nifedipine 20 mg/day) and methotrexate (10 mg/week) was initiated. Three years later (2017), a significant decline in pulmonary parameters was recorded: the forced vital capacity (FVC) decreased from 101% to 91% of predicted value, and DLCO (diffusing capacity for carbon monoxide) fell from 82.8% to 71.5%. High-resolution computed tomography (HRCT) of the chest revealed bilateral ground-glass opacities in the lower lobes, consistent with an NSIP pattern compatible with SSc-ILD, alongside prominent bilateral hilar lymphadenopathy with indeterminate etiology (Figure 1).

Laboratory investigations showed serum angiotensin-converting enzyme (ACE) levels at three times the upper normal limit. Although the patient declined invasive diagnostic procedures (bronchoscopy and mediastinoscopy), a presumptive diagnosis of stage I sarcoidosis was made. A course of low-dose prednisone 10 mg/day for 3 months was administered, resulting in the complete CT resolution of lymphadenopathy, so steroids were gradually tapered and stopped after 6 months.

Seven years after the initial onset (2021), the disease phenotype shifted to a rapidly progressive diffuse form. The modified Rodnan skin score (mRSS) increased from 15 to 32, accompanied by active digital ulcers, pitting scars, acro-osteolysis, and a transition to a “late” capillaroscopic pattern. Although dyspneea did not worsen and PFTs were quite stable (FVC 91%, DLCO 69%), thorax CT revealed complete resolution of hilar adenopathies but an extension of NSIP lesions (Figure 2).

Due to progressive skin involvement and the radiological extension of SSc-ILD, the immunosuppression regime was switched to mycophenolate mofetil (2 g/day), and bosentan (125 mg twice daily) was initiated for treating vasculopathy. The patient remained stable for the next 2 years.

The clinical course was further complicated in 2023 by an emergency admission for cardiac tamponade. Pericardiocentesis was performed, evacuating 1300 mL of serosanguinous fluid (negative for malignancy and infection). Due to a high risk of scleroderma renal crisis—driven by the diffuse skin subset, rapid mRSS progression, and anti-Scl70 positivity—high-dose glucocorticoids were avoided; the pericarditis was managed with non-steroidal anti-inflammatory drugs (ibuprofen 1200 mg/day for 2 weeks) and colchicine (1 mg/day for 4 weeks).

The next year (2024), despite non-significant variation in LFTs or worsening dyspneea, the follow-up HRCT revealed a fibrotic NSIP phenotype (honeycombing at the lung bases) (Figure 3). Still, persistent cutaneous activity with a high mRSS was noticed (mRSS = 31), so a multi-modal treatment escalation was implemented. The patient was started on off-label rituximab at 1 g every two weeks (two courses, six months apart) mainly for extensive skin involvement and nintedanib at 150 mg twice daily as an antifibrotic agent for SSc-ILD.

At the last follow-up (December 2025) the mRSS decreased (18), the patient had no digital ulcers, had class 2 dyspneea and stable PFTs (FVC 89%, DLCO 66%), and no significant abnormalities on echocardiography were noticed.

### 3.2. Case 2

A 40-year-old male, who was an active smoker and a construction worker, presented in 2020 with a subacute onset of cough, exertional dyspnea, night sweats, and weight loss. Initial laboratory investigations revealed mild leukocytosis (14,300/mmc) and a non-specific inflammatory syndrome (ESR = 60 mm/h, CRP = 27 mg/L). Despite multiple courses of empirical antibiotic therapy, his symptoms persisted. Initial PFTs were near-normal (FVC 91%, FEV1 80%). HRCT of the chest identified enlarged mediastinal and hilar lymph nodes, scattered bronchocentric micronodules (1–5 mm) in the mid-to-upper zones, and subpleural ground-glass opacities (Figure 4). Many of the radiological abnormalities were pointing to sarcoidosis, so the pneumologist decided to continue investigations. Bronchoalveolar lavage (BAL) showed normal cellular count with slight lymphocytosis (15%), and a surgical wedge resection of a left lower-lobe micronodule revealed only fibrosclerosis and anthracosis. At that moment, no specific treatment was recommended by the pneumologist.

One year later (2021), the patient developed acroparesthesia, inflammatory arthralgias, and skin thickening of the fingers, hands, and face (Figure 5A). Immunological profiling confirmed high titers of anti-Scl70 antibodies (>200 UI/mL), and nailfold capillaroscopy showed an “early” scleroderma pattern. A diagnosis of systemic sclerosis with limited cutaneous involvement was established based on the ACR/EULAR 2013 classification criteria for SSc [26]. Radiographic incidental findings included a spotted bone pattern in the lower limbs, which is characteristic of osteopoikilosis (Figure 5B). Treatment was initiated with methotrexate (20 mg/week) and low-dose prednisone (10 mg/day).

Despite the above mentioned therapy, after 6 months (March 2022) PFTs demonstrated a decline to moderate restriction (FVC 59%, DLCO 66%). Repeated BAL showed significant lymphocytosis (23%), and HRCT revealed persistent nodular infiltrates with emerging ground-glass opacities in the lower lobes. Following the self-discontinuation of glucocorticoids (GC), 18 months later (September 2023), the patient experienced a severe flare characterized by painful red-blue subcutaneous nodules, worsening dyspnea, and a marked inflammatory syndrome (CRP 63 mg/L). Follow-up CT (Figure 6) showed a significant increase in the size and number of paratracheal and hilar nodules. At this stage, serum ACE levels rose to five times the upper normal limit, raising the probability for the diagnosis of stage II sarcoidosis complicating the underlying SSc.

Glucocorticosteroids were restarted with prednisone 20 mg/day for 3 months then tapered to 5 mg/day. Given the dual progression of ILD and skin involvement (mRSS =11 in 2021, 15 in March 2022, then 21 in September 2023), the immunosuppressive regimen was switched to mycophenolate mofetil 2 g/day.

Under this regimen, the patient achieved stabilization of lung function (FVC = 56%, DLCO = 64%), partial regression of the mediastinal lymphadenopathy and NSIP lesions, improvement of skin involvement (mRSS = 7) at the two year follow-up (December 2025).

### 3.3. Case 3

In July 2021, a 63-year-old male who was a non-smoker with no relevant occupational exposure presented with persistent fever, dyspnea on mild-to-moderate exertion, cough, and joint swelling/arthralgia. HRCT of the chest revealed a bilateral reticulo-micronodular interstitial pattern accompanied by bilateral mediastinal and hilar lymphadenopathy (Figure 7).

Laboratory findings indicated an elevated inflammatory syndrome (ESR = 74 mm/h, CRP = 44 mg/L) with a slightly increased ACE level above upper normal level. Initial bronchoscopy and bronchoalveolar lavage (LBA) were negative for malignancy and tuberculosis (non-specific cytology, negative cultures). Based on these findings, a biopsy-proven diagnosis of stage II mediastino-pulmonary sarcoidosis was established. Systemic corticosteroid therapy (methylprednisolone starting with 32 mg/day) was initiated with good response at 3 months (no fever, dyspneea and cough improvement, regression of hilar adenopathies on HRCT follow-up). Steroids were gradually tapered in the next 3 months to 8 mg methylprednisolone/day but were discontinued after six months due to the development of a gastric ulcer.

In June 2022, the patient experienced a recurrence of fever, dyspnea, and polyarthralgia. HRCT imaging confirmed the return of the reticulo-micronodular pattern and mediastinal adenopathy. While transbronchial biopsy was inconclusive, a mediastinoscopy with lymph node biopsy was performed, providing histopathological confirmation of sarcoidosis (noncaseating granuloma). Oral corticosteroid therapy (mehtylprednisolone 32 mg) was reintroduced for a 12-month course. The patient’s symptoms improved and lung function tests remained stable, but steroid tapering to 16 mg methylprednisolone/day was associated with inflammatory arthralgias of the small joints of the hands; methotrexate up to 20 mg/week was added to the treatment regime so corticosteroid dose could be reduced to 8 mg methylprednisolon/day.

Between December 2023 and February 2024, the patient developed new clinical features, including sclerodactyly and Raynaud’s phenomenon. Serological testing revealed positive antinuclear antibodies (ANAs) and strongly positive anti-Scl-70 antibodies. Nailfold capillaroscopy demonstrated an “early” scleroderma pattern. HRCT revealed a change to an NSIP pattern with bilateral ground-glass opacities distributed predominantly in the middle and lower lobes, primarily in the subpleural regions, and thickening of the interlobular and intralobular septae, creating a net-like appearance and traction bronchiectasis (Figure 8).

A multidisciplinary review (pulmonology, rheumatology, and radiology) based on the ACR/EULAR 2013 classification criteria for SSc [26] established that the patient had diffuse cutaneous SSc with associated ILD (SSc-ILD) exhibiting a fibrotic NSIP pattern. Due to the presence of several risk factors (male sex, age > 60, diffuse cutaneous form, and Scl-70 positivity) for progression of SSc-ILD, a dual-action therapeutic regimen was initiated: mycophenolate mofetil 2000 mg/day and mintedanib (150 mg twice daily). Longitudinal PFTs showed relative stability: FVC: 2.68 L (65%) vs. 2.54 L (63%); DLCO: 19.73 mL/min/mmHg (71%) vs. 18.51 mL/min/mmHg (66%), and six-minute walk test (6 MWT) distance of 580 m without desaturation.

Despite the initial good follow-up, the evolution was marked mostly by progressively severe visceral involvement related to systemic sclerosis in the next two years. In May 2025, the capillaroscopic pattern progressed to a late pattern associated with multiple digital ulcers (bosentan 125 mg twice daily was added to the treatment). In September 2025, two sessions of endoscopic balloon dilation were performed for tight pyloro-bulbar stenosis. Despite a stable fibrotic NSIP pattern on HRCT (Figure 9), lung function tests continued to decline (October 2025 FVC = 2.1 L, DLCO = 13.9 mL/min/mmHg.

Longitudinal monitoring in December 2025 also revealed heart involvement, with the patient first developing rhythm and conduction disturbances (grade I atrioventricular block, premature ventricular beats, and recurrent paroxistic supraventricular arrhythmias). NT-proBNP levels continued to increase over the next couple of months along with a slight stable elevation of troponin levels (two times upper normal limit); echocardiography showed a significant decrease in left and right ventricle systolic function (left ventricle ejection fraction 45%, right ventricle ejection fraction 27%), large pericardial effusion, and mild pulmonary hypertension (sPAP = 42 mmHg). Cardiac MRI showed slight dilation of the right ventricle with moderate systolic dysfunction, focal intramyocardial fibrosis mainly in the anterior and basal part of the interventricular septum and a very slightly elevated T2 sequence values suggestive of diffuse myocardial edema. Considering the aggressive evolution of SSc with extensive skin involvement (mRRS 11 in February 2024 increased to 31 in December 2025), progressive SSc-ILD, probably primary heart involvement related to SSc, the multidisciplinary decision was to start off-label rituximab 1 g iv 2 weeks apart. The patient has not been reevaluated yet after the first course of rituximab.

## 4. Discussion

Sarcoidosis and systemic sclerosis are both multisystem disorders of unknown etiology. Sarcoidosis manifests as a noncaseating granulomatous disease most commonly affecting the lungs, skin, and eyes, while systemic sclerosis is a connective tissue disorder characterized by cutaneous sclerosis, visceral fibrosis, and vasculopathy. The incidence of sarcoidosis is 50 to 400 per million per year [27] and the incidence of systemic sclerosis is 20 per million per year, so the likelihood of an overlap disease is expected to be very low [28,29].

A comprehensive review of the literature, conducted through the PubMed database using the keywords “systemic sclerosis” (AND) “sarcoidosis”, identified 25 relevant articles reporting cases of overlap syndrome between these two conditions, with a total of 36 patients [30,31,32,33,34,35,36,37,38,39,40,41,42,43,44]. Only articles published in English were included to ensure clarity and consistency in data extraction and interpretation. Studies, case reports, case series, and reviews discussing patients diagnosed with both systemic sclerosis and sarcoidosis, either simultaneously or sequentially, were included. Articles not related to clinical cases or clinical data (e.g., purely experimental or animal studies), studies without clear diagnostic criteria or insufficient clinical details to confirm overlap or non-English language publications were excluded. An overlap case was defined as a patient who fulfills established classification criteria for systemic sclerosis and has documented clinical, radiologic, or histopathologic evidence of sarcoidosis (presence of noncaseating granulomas, typical organ involvement). Overlap includes both concurrent diagnosis and sequential development of one disease after the other. If specific clinical or diagnosis data were unavailable, such cases were included only if the overlap diagnosis was explicitly stated. Otherwise, incomplete cases were excluded from detailed analysis.

This association appeared more frequently in females, as evidenced by a female-to-male ratio of 2.63:1; the mean age at diagnosis was 53.64 years, based on the 26 cases where age was available, with male patients having a mean age of 41.8 years and female patients having a mean age of 57.2 years at diagnosis. Regarding the temporal sequence of disease onset, systemic sclerosis preceded sarcoidosis in 17 cases (47.22%), sarcoidosis was diagnosed before systemic sclerosis in 13 cases (36.11%), and both diseases were diagnosed concurrently in 6 patients (16.66%). As for the subtype of systemic sclerosis, the limited cutaneous form, limited SSc was roughly twice as common as diffuse SSc in this overlap though this information was not specified in some cases. Six patients had anti-centromere antibodies, nine had anti-Scl-70 antibodies, and six had various other autoantibodies, including Ro, Ka [30], TIF-1 γ [31], U1-RNP [32], and AMA-2 [33], while two patients tested negative for antinuclear antibodies [34], and two articles did not mention immunological profiles. Pulmonary involvement was present in 26 patients and was attributed either to sarcoidosis, systemic sclerosis, or both conditions. Sarcoidosis manifested as bilateral hilar lymphadenopathy in 17 cases and as upper-lobe ILD in 11, while systemic sclerosis-related lung involvement appeared in 14 patients as ILD characterized by honeycombing and ground-glass opacities. Notably, no cases were reported without any pulmonary involvement.

Given the extensive time frame covered by these case reports, ranging from 1979 to 2025, therapeutic approaches varied widely, with corticosteroids being the most commonly administered treatment, used in 12 patients either alone or in combination with immunosuppressive agents such as azathioprine (three cases) [35,36,37], mycophenolate mofetil (one case) [38], and cyclophosphamide (one case) [39]; treatment data were not available in nine articles.

It is important to note that several articles lacked complete clinical data, with age being unreported in two cases [40,41], sex in another two [41,42], pulmonary involvement unspecified in two cases [40,41], systemic sclerosis subtype omitted in three reports [32,40,41], autoantibody profiles absent in two [40,41], and treatment details not provided in eight of the articles reviewed [32,33,41,42,43].

Each of the three cases presented herein has certain particularities.

The first case is a young female with the onset of scleroderma before sarcoidosis, similar to most of the cases reported in the literature. Although sarcoidosis was not confirmed on biopsy of the lymph nodes, high ACE levels and the prompt response of the mediastinal adenopathies to a short corticosteroid, in the absence of SSc-ILD involvement and other causes for sarcoid-like reaction, led the pneumologist to the presumed diagnosis of stage 1 sarcoidosis. Regarding the SSc evolution, we noticed exacerbation of skin thickening in the late stage, along with progression of the ILD and cardiac tamponade despite immunosuppression treatment with mycophenolate.

The second case is a male patient with the onset of sarcoidosis before systemic sclerosis, also similar to most of the cases reported in the literature. Although the initial HRCT was highly suggestive of sarcoidosis (showing both hilar adenopathy, micronodules, and interstitial changes), the diagnosis was initially missed because the ACE levels were normal and lymph node biopsy was not typical. Along with that, the patient was an active smoker and construction worker, which increased the possibility of pneumoconiosis or mixed-dust pneumoconiosis—a condition that can mimic sarcoidosis both radiologically and clinically. The diagnosis of probable sarcoidosis was revealed by the presence of erythema nodosum lesions associated with high ACE levels after glucocorticoids were tapered. At that moment, pneumologist ruled out occupational lung disease (the patient was already retired), hypersensitivity pneumonitis, infection, lymphoma, and drug-induced adenopathy. With the onset of systemic sclerosis, the pattern of lung involvement changed, associating the nodular ILD pattern with that of cellular NSIP, and involving both the upper and lower lobes. The therapeutic challenge in choosing the appropriate immunosuppression regime was to decide which of the two autoimmune diseases was predominant in the lungs. We also closely monitored creatine levels (monthly) and hypertension (twice/week) due to corticosteroid use, as the patient had several risk factors for scleroderma renal crisis (male, presence of anti-topo1 antibodies). Another major particularity is the association of osteopoikilosis.

In the third case, the diagnosis of sarcoidosis was confirmed by lymph node biopsy. Initially, the patient had only mediastinal involvement with good response to steroids, but the patient experienced a flare after stopping steroids due to side effects, along with interstitial involvement. Systemic sclerosis onset occurred while the patient was treated with steroids and MTX for sarcoidosis, and despite that, the evolution of SSc was very aggressive with rapidly skin involvement, digital ulcers, and musculoarticular, gastrointestinal, lung, and heart involvements. We consider the lung involvement to be related to both diseases, with continuous progression despite treatment with immunosuppressive drugs, corticosteroids, and antifibrotics. The heart involvement was also problematic, as it could have been related to scleroderma but also to sarcoidosis, albeit rare [44].

The three cases presented herein illustrate the clinical heterogeneity and intricate temporal relationship between systemic sclerosis and sarcoidosis. While both are multisystemic diseases characterized by immune dysregulation, their coexistence creates unique diagnostic and therapeutic challenges.

### 4.1. Temporal Patterns of Onset

The chronological appearance of the two diseases in our case series reflects the variability documented in the literature.

Case 1 followed the more frequent pattern where SSc precedes sarcoidosis. Although histological confirmation was not obtained, the clinical triad of high ACE levels, mediastinal lymphadenopathy, and a prompt response to low-dose corticosteroids strongly supports the diagnosis of stage I sarcoidosis.

Cases 2 and 3 demonstrated the opposite sequence (sarcoidosis preceding SSc). In case 2, the diagnosis was initially elusive due to the normal ACE levels and a non-specific initial biopsy (anthracosis). The diagnosis was only unmasked by a flare—manifesting as erythema nodosum and significantly elevated ACE levels—following glucocorticoid tapering.

### 4.2. Pulmonary Involvement: A Hybrid Challenge

Determining the predominant driver of lung pathology is a central challenge.

In case 1, ILD was considered to be related to SSc and was treated with MMF. The patient fulfilled only the radiological criteria for progressive fibrotic ILD, but because she was anti-Scl70-positive as a risk factor for progression, nintedanib was initiated for SSc-ILD.

In case 2, the lung imaging showed an evolution into a hybrid pattern, combining the bronchocentric micronodules of sarcoidosis with the cellular NSIP pattern of SSc. This necessitated a careful balance of immunosuppression to address both granulomatous and fibrotic processes.

In case 3, the lung involvement was particularly refractory, with continuous progression despite combined treatment with immunosuppressants, corticosteroids, and antifibrotics (that were started early for SS-ILD with risk factors for progression), suggesting a synergistic detrimental effect of the two diseases.

### 4.3. Cardiac Management

Cases 1 and 3 both involve serious cardiac issues, including pericardial tamponade, arrhythmias, ventricular dysfunction, rising NT-proBNP levels, and mild pulmonary hypertension. These are important clinically and may be among the most notable features of this series, as cardiac tamponade has not been systematically described in the published SSc–sarcoidosis overlap literature. Cardiac MRI, FDG-PET or right-heart catheterization were not performed for case 1, but the temporal sequence and the occurrence of tamponade during the remission period of sarcoidosis make it unlikely that sarcoidosis is the cause of the tamponade. In case 3, the attribution of cardiac involvement to SSc or sarcoidosis was even more challenging. Cardiac MRI mainly showed intramyocardial fibrosis and predominantly right ventricle involvement, so we think that the patient had primary heart involvement related to SSc.

### 4.4. Renal Safety

In all cases, the use of glucocorticoids (GCs) required vigilant monitoring. For case 2, the combination of male sex, anti-Scl70 antibodies, and GCs use created a high-risk profile for scleroderma renal crisis (SRC), necessitating strict control of blood pressure twice/week and creatinine levels monthly.

### 4.5. Unique Phenotypic Features

Case 2 is particularly noteworthy due to the association with osteopoikilosis, a rare bone dysplasia. While generally benign, its presence alongside an SSc–sarcoidosis overlap adds to the rarity of this clinical triad.

Case 3 represents a highly aggressive phenotype, with the onset of SSc occurring while the patient was already receiving steroids and methotrexate. This case demonstrated rapid multisystemic deterioration (gastrointestinal, digital ulcers, cardiac involvement), suggesting that sarcoidosis might, in some instances, precede or even mask a particularly malignant form of SSc.

## 5. Conclusions

The coexistence of systemic sclerosis (SSc) and sarcoidosis represents a rare overlap that challenges conventional diagnostic and therapeutic paradigms. Clinicians must maintain a high index of suspicion for sarcoidosis when atypical hilar lymphadenopathy or elevated ACE levels appear in SSc patients. Conversely, an initial diagnosis of sarcoidosis should not preclude monitoring for systemic autoimmune features, as respiratory symptoms can precede skin involvement by years. Managing this overlap requires a delicate balance. While sarcoidosis often demands the use of glucocorticoids, the inherent risk of scleroderma renal crisis—exacerbated by high-dose steroids and anti-Scl70 positivity—necessitates the use of steroid-sparing agents. Success in managing such patients depends on a coordinated effort between rheumatologists, pulmonologists, and cardiologists to navigate life-threatening complications. In summary, the SSc–sarcoidosis overlap is a high-risk phenotype. Early recognition of the coexistence of SSc and sarcoidosis may identify a subgroup that requires particularly careful multidisciplinary evaluation, because pulmonary, cardiac, and therapeutic risks may overlap and complicate standard management.

## Figures and Tables

**Figure 1:**
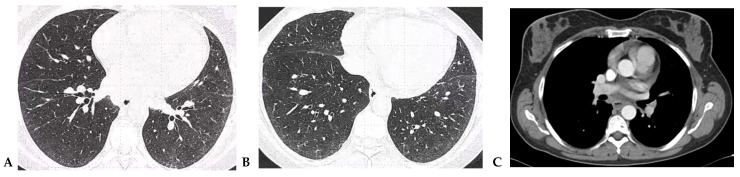
Initial lung CT (2017) of the first case, showing ground-glass opacities distributed predominantly in the lower lobes in the subpleural regions, and thickening of the interlobular and intralobular septae with traction bronchiectasis (non-specific interstitial pneumonia) (**A**,**B**) linked to SSc-ILD alongside mediastinal adenopathies (**C**) with indeterminate etiology.

**Figure 2:**
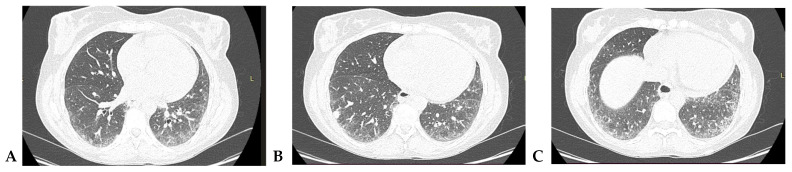
Follow-up lung CT (2021) of the first case, showing an extension of ground-glass opacities distributed in the middle and lower lobes (**B**,**C**), particularly in the subpleural regions, and thickening of the interlobular and intralobular septae (**A**,**B**) with traction bronchiectasis compatible with SSc-ILD, but significant mediastinal adenopathies were not detected.

**Figure 3:**
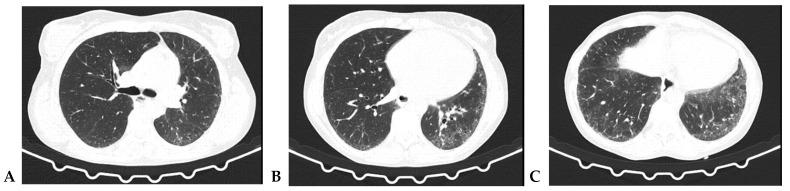
HRCT of the first case 10 years after the SSc diagnosis, showing an extension of ground-glass opacities distributed in the middle (**B**) and lower lobes (**C**), particularly in the subpleural regions, thickening of the interlobular and intralobular septae with traction bronchiectasis (**A**,**B**), and honeycombing in the bases of the lung (**C**) (progressive fibrotic SSc-ILD). There were no significant mediastinal adenopathies.

**Figure 4:**
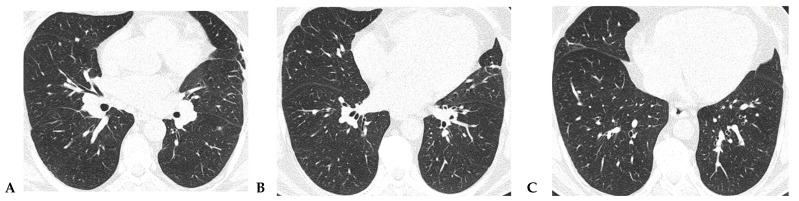
Thorax CT of the second case (2020), showing roughly symmetrical bronchocentric micronodular infiltrate (**A**). In more central areas, there is dense parenchymal opacification caused by the conglomeration of nodules around the bronchovascular structures (**B**,**C**).

**Figure 5:**
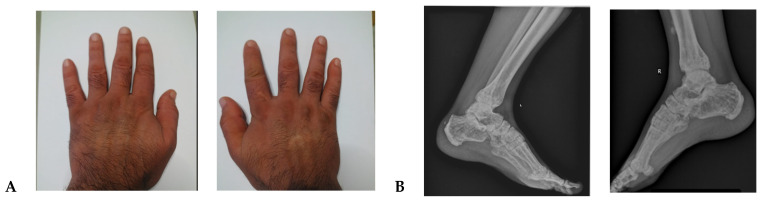
(**A**) Images showing the patient’s puffy hands with thickening of the skin in the fingers and dorsal part of the hands. (**B**) Circumscribed sclerotic areas near the ends of the tibia and perineum (osteopoikilosis). L—left foot, R—right foot.

**Figure 6:**
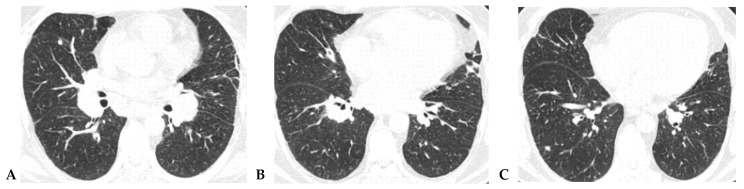
Follow-up thorax CT of the second case (2022), showing symmetrical bronchocentric micronodular infiltrate (**A**), dense parenchymal opacification caused by the conglomeration of nodules around the bronchovascular structures (**B**,**C**), and mediastinal adenopathies (**A**). All the radiological features described were more likely related to sarcoidosis rather than SSc-ILD.

**Figure 7:**
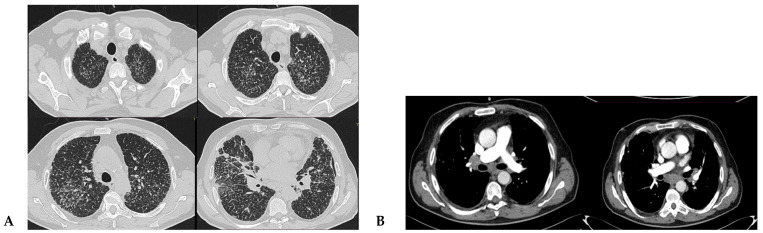
First thorax CT of the third case (2021) showing diffuse, roughly symmetrical distribution of micronodules throughout the lung parenchyma with a bronchocentric pattern, which is particularly noticeable in the mid and upper zones (**A**); in more central areas, there is dense opacification where these nodules have conglomerated around the bronchovascular structures along with mediastinal adenopathies (**B**). Initial CT findings reveal typical radiological features of stage II sarcoidosis.

**Figure 8:**
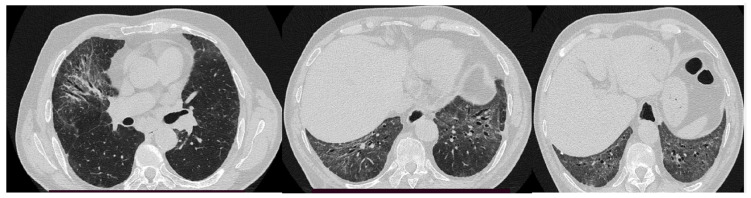
Follow-up CT of the third case (February 2024), showing significant regression of previously described micronodules/conglomerate nodules throughout the mid and upper zones lung parenchyma, regression of mediastinal adenopathies compatible with sarcoidosis diagnosis; instead, bilateral ground-glass opacities distributed predominantly in the middle and lower lobes mainly in the subpleural regions, and thickening of the interlobular and intralobular septae and traction bronchiectasis are noticed pointing to SSc-ILD.

**Figure 9:**
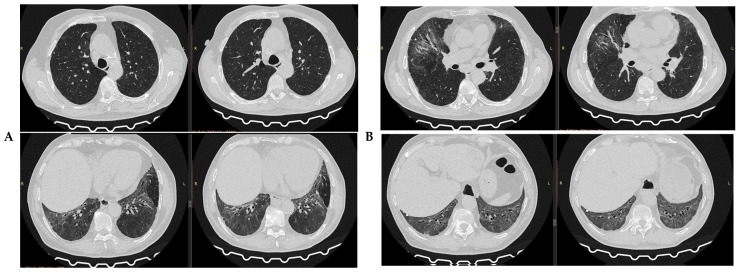
Comparable HRCT evolution after antifibrotic treatment initiation (**A**—NSIP pattern at SSc-ILD onset; **B**—12 months after immunosuppression with mycophenolate and nintedanib).

## Data Availability

The data presented in this study are available on request from the corresponding author due to institutional policies governing the security and confidentiality of patient databases at Sfanta Maria Clinical Hospital.

## Abbreviations

SSc	Systemic sclerosis
SSc-ILD	Systemic sclerosis interstitial lung disease
PFTs	Pulmonary functional tests
ILD	Interstitial lung disease
CT	Computed tomography
HRCT	High-resolution computerized tomography
NSIP	Non-specific interstitial pneumonia
mRSS	Modified Rodnan skin score
BAL	Bronchoalveolar lavage
ACE	Angiotensin-converting enzyme
GC	Glucocorticoids
MMF	Mycophenolate mofetil
6 MWT	6 min walking test
MRI	Magnetic resonance imaging
NT proBNP	N-terminal pro b-type natriuretic peptide
FDG-PET	Fluorodeoxyglucose–positron emission tomography
